# Supplementary material for: Atomic-scale manipulation of single-polaron in a two-dimensional semiconductor
Source: Nat Commun. 2023 Jun 21;14:3690. doi: 10.1038/s41467-023-39361-0 (PMC10284845; doi:10.1038/s41467-023-39361-0)
Supplement: Supplementary file 1 — Supplementary information [file 41467_2023_39361_MOESM1_ESM.pdf]

Supplementary Information for

Atomic-scale Manipulation of Single-Polaron in a Two-Dimensional Semiconductor

Huiru Liu[1,2†], Aolei Wang [3†], Ping Zhang[1,2], Chen Ma[1,2], Caiyun Chen[1,2], Zijia Liu[1,2,4], Yi-Qi Zhang[1,2], Baojie Feng[1,2,5], Peng Cheng[1,2], Jin Zhao [3,6,7,8*], Lan Chen[1,2,4*], Kehui Wu[1,2,4,5*]

[1] *Institute of Physics, Chinese Academy of Sciences, Beijing 100190, China*

[2] *School of Physical Sciences, University of Chinese Academy of Sciences, Beijing, 100190, China*

[3] *Department of Physics, University of Science and Technology of China, Hefei, Anhui 230026, China*

[4] *Songshan Lake Materials Laboratory, Dongguan, Guangdong, 523808, China*

[5] *Interdisciplinary Institute of Light-Element Quantum Materials and Research Center for Light-Element Advanced Materials, Peking University, Beijing 100871, China.*

[6] *ICQD/Hefei National Research Center for Physical Sciences at the Microscale, University of Science and Technology of China, Hefei, Anhui 230026, China*

[7] *Department of Physics and Astronomy, University of Pittsburgh, Pittsburgh, 15260, Pennsylvania, USA*

[8] *Hefei National Laboratory, University of Science and Technology of China, Hefei, Anhui 230026, China*

[†] These authors contributed equally to this work.

*Emails: khwu@iphy.ac.cn (K.W.); lchen@iphy.ac.cn (L.C.); zhaojin@ustc.edu.cn (J. Z.)

This PDF file includes:

Supplementary Note. I Electrostatic field simulation of surface potential

Supplementary Note. II The formation of the ring-like feature in the d*I*/d*V* maps

Supplementary Note. III *Ab initio* Simulation: Methodology and Results

Supplementary Note. IV Hopping of polarons

Supplementary Note. V Additional experiments and data

Supplementary Note. VI Discussion of possible bi-polaron or polaron clustering

Supplementary Figure 1 to 21

Supplementary Table 1

Supplementary References

Supplementary Note. I Electrostatic field simulation of surface potential

The STM image of polarons is dominated by the electrostatic field associated with the trapped electron. Therefore, the electrostatic field is simulated and compared with the STM images. Considering a semi-classical structural model, $CoCl_2$/HOPG system can be simplified to a film with thickness $L = 0.58$ nm and dielectric constant ε_{CoCl2} sandwiched between a HOPG substrate and vacuum. To mimic the charged polaron, a point charge was introduced at a distance d from the surface of $CoCl_2$. In order to simplify the calculations, we treat the HOPG as an ideal metal substrate to bring in the screening effect, which may overestimate the actual screening effect. According to this hypothesis, the surface potential created by a local charge on the $CoCl_2$ layer can be calculated using the multiple image charge technique [1] as illustrated in Supplementary Fig. 1a. The total potential at the vacuum/$CoCl_2$ interface is equal to the sum of all individual image charges. This method has been previously performed to study similar systems [2-4]. For the type-II polaron with one charge on single cobalt atom (Supplementary Fig. 1b), the expression of the potential at the layer surface, $U_{1s}(r)$, is:

$$U_{1s} = \frac{q}{4\pi\varepsilon}\frac{1}{\sqrt{r^2+d^2}} + \frac{q}{4\pi\varepsilon}\times\sum_{n=1}^{\infty}(-1)^n\gamma^{n-1}\left\{\frac{1}{\sqrt{r^2+(4nd-d)^2}}+\gamma\frac{1}{\sqrt{r^2+(4nd+d)^2}}\right\} \quad (1)$$

where q is the charge of polaron, r is the distance to the origin of coordinates of surface. ε is the effective dielectric constant of vacuum/$CoCl_2$ interface, $\gamma = \frac{\varepsilon_{cocl2}-\varepsilon_{vac}}{\varepsilon_{cocl2}+\varepsilon_{vac}}$, $\varepsilon_{vac} = 1$, $\varepsilon_{CoCl2} = 20$ (referring to the typical value of TMD [1]). In the case of the type-I polaron with one charge on three cobalt atoms, the surface potential is the superposition of contribution from three point-charges with 1/3 q and distance of atomic period $a = 3.54$ Å between them (Supplementary Fig. 1c). The corresponding expression is:

$$U_{3s} = \frac{q}{4\pi\varepsilon}\frac{1}{3}\left(\frac{1}{\sqrt{r_1^2+d^2}}+\frac{1}{\sqrt{r_2^2+d^2}}+\frac{1}{\sqrt{r_3^2+d^2}}\right)+\frac{q}{4\pi\varepsilon}\times \frac{1}{3}\sum_{n=1}^{\infty}(-1)^n\gamma^{n-1}\sum_{i=1}^{3}\left\{\frac{1}{\sqrt{r_i^2+(4nd-d)^2}}+\gamma\frac{1}{\sqrt{r_i^2+(4nd+d)^2}}\right\} \tag{2}$$

Since the charge in polaron is locate at cobalt atoms, we assumed the charge is in the middle of the layer, $d = L/2 = 0.29$ nm. Considering the high repeatability of a single operation, we set the charge q equal to the amount of charge per electron, $q = e = -1.6 \times 10^{-19}$ C. The order of screening is up to $n = 1000$. Then the surface band bending due to the electrostatic potentials are calculated to be eU_s.

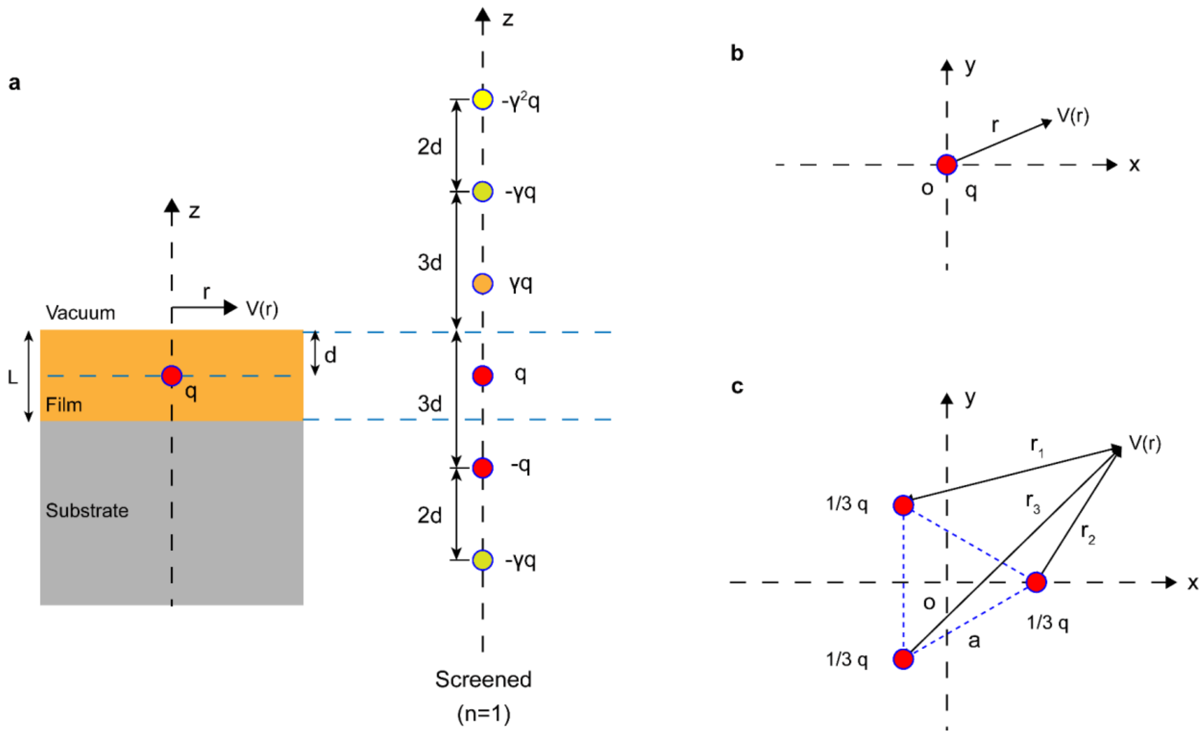

Supplementary Fig. 1: Electrostatic field simulation of surface potential. (a) Schematic drawing of multiple image charge technique. The charge located in the $CoCl_2$ film at a distance d from its surface is represented with the red dot. The orange yellow and milky dots show sequential images of the charge reflected at the vacuum/$CoCl_2$ and $CoCl_2$/HOPG interfaces. The diagram only shows the first order of screening. **(b)** Schematic drawing of charge distribution of type-II polaron on the plane paranal to interface.

The charge q is located at origin of 2D coordinate. **(c)** Schematic drawing of charge distribution of type-I polaron projected on the plane paranal to interface. The charges with 1/3 q are evenly distributed on three vertices of equilateral triangle with length of side a = 3.54 Å.

Supplementary Figure 2 shows the result of the simulated electrostatic potential. The difference in charge distribution between two types of polarons give the depleted potential area with diameters of 3.26 nm and 2.90 nm, respectively, which are qualitatively in good agreement with the experiments. The smaller differences compared with the experiments may come from overestimation of the screening effect of HOPG substrate in simulations. Importantly, the potential sizes are in line with the usual effective Coulomb field (a few nanometers [5]) of the isolated electron in semiconductor, suggesting a single polaron is hosted in each ring feature.

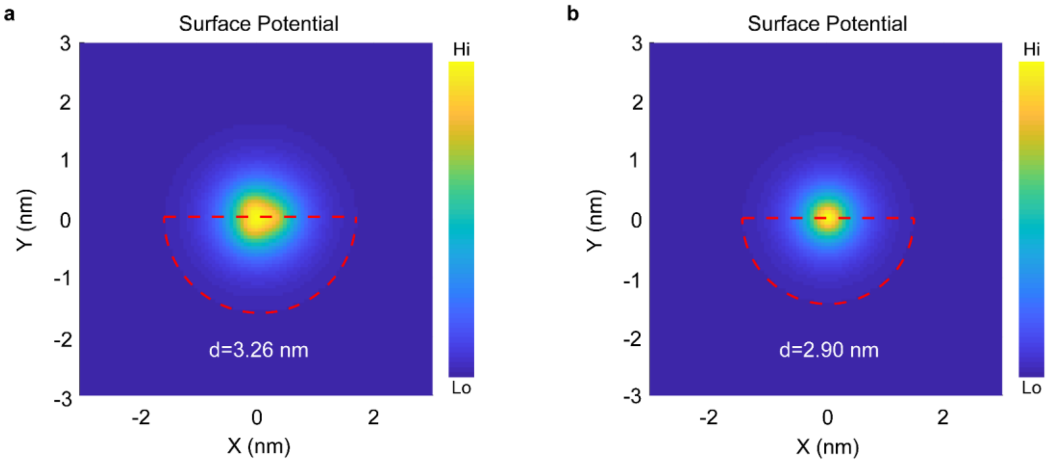

Supplementary Fig. 2: Simulated surface electrostatic potential diagrams of the charge distribution around type-I (a) and type-II (b) polarons. The solid red fan frames mark 1/2 circle, and the corresponding diameters are labelled. “Hi” and “Lo” in the color scales are the abbreviation of “High” and “Low”.

Supplementary Note. II The formation of the ring-like feature in the d*I*/d*V* maps

Our STM image and dI/dV map of polarons in $CoCl_2$ exhibit depressed feature and a ring-like feature with depressed inner region, respectively in empty state images. The ring-like feature appears similar as the ring feature observed in switchable charged defect states [6-9]. However, a polaron is a static trapped charge. Therefore, it is necessary to clarify the similarity and difference between a switchable charged defect state and a static charged state of polaron.

In the case of a switchable charge state, the electric field from STM tip induces surface band-bending (TIBB). When the tip approaches to the defect from a far distance, the defect will be charged when the defect level comes cross the Fermi level, thus instantaneously opening a new tunneling channel, as characterized by a sharp peak in dI/dV curve and a ring with sharp edge in dI/dV map [6].

However, in the case of a static charged objective on the surface, the dominant feature in STM image is the band-bending induced by the electrostatic field from the charge center (charge-induced band bending), which modifies the density of states (DOS) of the surface around the defect [1]. Such kind of band bending is also observed in ferroelectric domains on the surface [10]. In this case, the tip-induced band-bending (TIBB) is negligible if it does not induce change of the charge state.

For comparison, we did not observe any charging peak in our dI/dV curves and line maps, but only the bending of conductance band at the position near the polaron (see Fig. 3c of main text). In fact, when we measured the dI/dV map in constant-height mode, we cannot observe the ring feature with bright edge but a dark pit around the polaron, as shown in Supplementary Fig. 3b. The main reason for the difference is because in the constant height mode, the STM tip is kept at the same distance from the surface, thus the TIBB is homogeneous everywhere and can be neglected. The dominant feature in STM image is then the band-bending induced by the electrostatic field from the charge center, which modifies the surface DOS at the

tunneling bias. A negative charge center will result in upper bending of the CBM, leading to lower DOS contributions under a certain sample bias and thus lower current in empty state images, in good agreement with the STM image and d*I*/d*V* maps in constant height mode. It can also be equivalently described by decreased effective tunneling bias V_{eff}, a modified sample bias by charge potential as shown in Supplementary Fig. 3e.

On the other hand, the constant current mode is more complicated. In this case, the height of the STM tip is instantaneously adjusted to maintain a constant tunneling current, therefore the TIBB cannot be neglected, but has to be convoluted in both the STM image and d*I*/d*V* maps. Qualitatively, when the STM tip approaches closer to the polaron during scanning, the DOS on surface decreases due to the polaron-induced band-bending (PIBB) as mentioned above. In order to maintain a constant current, the tip is brought to a closer distance to the surface. This will then result in a stronger TIBB, and an increase (decrease) of the surface DOS from conduction (valence) band. Therefore, in a proper range the measured DOS would reach a maximum, and thus generates a ring-like feature in the d*I*/d*V* map.

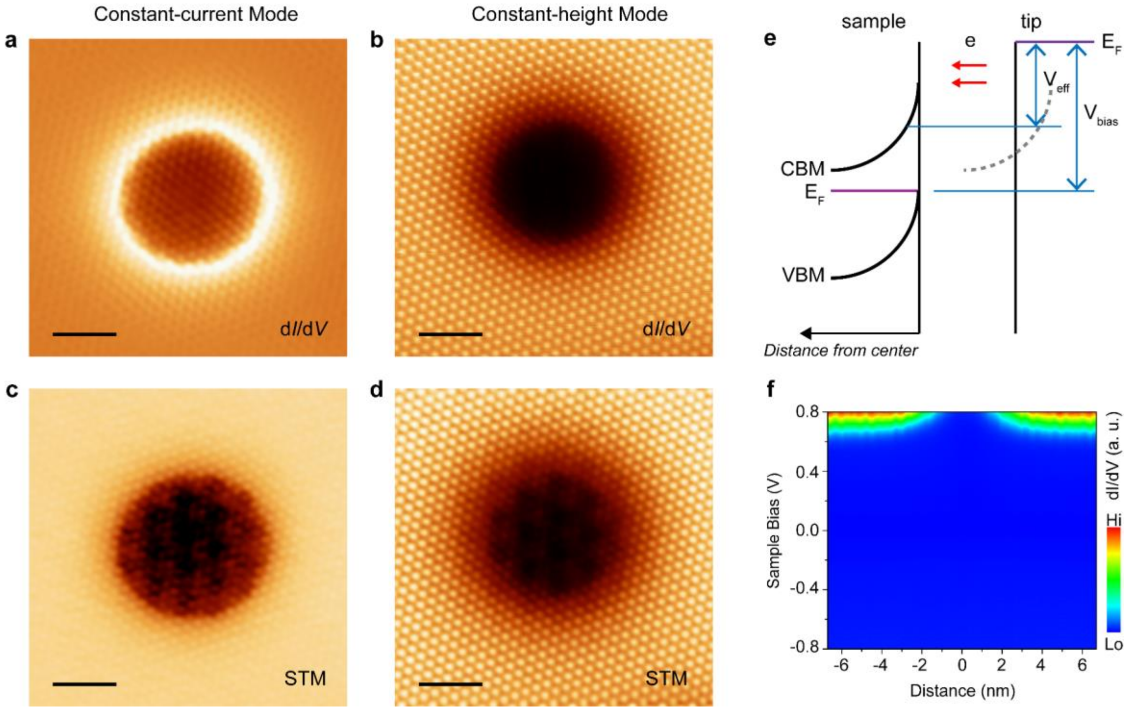

Supplementary Fig. 3: Characterization of polaron under constant- current and height mode. (**a-d**) Comparison of the depression feature in constant current STM image and d*I*/d*V* maps with those in constant height STM mode (V_s = 750 mV, I = 10 pA, V_{mod} = 20 mV). Scale bars: 2 nm. (**e**) is the energy diagram at the tunneling gap. V_{bias} and V_{eff} are the bias directly applied onto sample and effective bias applied between tunneling junction.

Based on the electrostatic field simulation of polaron in the Part. I, an extra contribution from the potential curve induced by the tip height change during scanning under constant-current mode can be estimated as follow. The total potential at the vacuum/$CoCl_2$ interface is equal to the sum of PIBB and TIBB. For a qualitative understanding taking type-II polaron as example, the spatial potential distribution of a charged center is inversely proportional to the distance *r* between the tip position and the charged center, thus simplistically applied to the top of STM tip. The total potential:

$$U_{1t} = U_{1s} + (-1) \times \lambda \times \frac{1}{\sqrt{r^2 + D^2}} \quad (3)$$

Where "-1" means the opposite direction of tip potential under positive sample bias with respect to the charge potential in sample. λ is the factor of relative strength, which is related to the field strength of tunneling junction. Assuming the tunneling gap is D = 1 nm. From Fig. 3c, the larger bias V_s, the smaller and deeper pit, thus the smaller tip height and the stronger field of tunneling junction under constant-current mode, corresponding to the larger λ value. Supplementary Figure 4 shows the change of surface potential with the increase λ. When λ =0, only PIBB is concluded. The curve shows a monotonous line shape, which perfectly agree with the dark pit feature in the d*I*/d*V* map at constant-height mode. With the increasing of λ, as well as sample bias V_s, two shoulder peaks appear symmetrically, marked by red lines. It

can be found that the distance between shoulder peaks gradually shrinks and the depth gets shallower with increasing λ or V_s, which are also consistent with our observation (the right panels of Fig. 3a in main text).

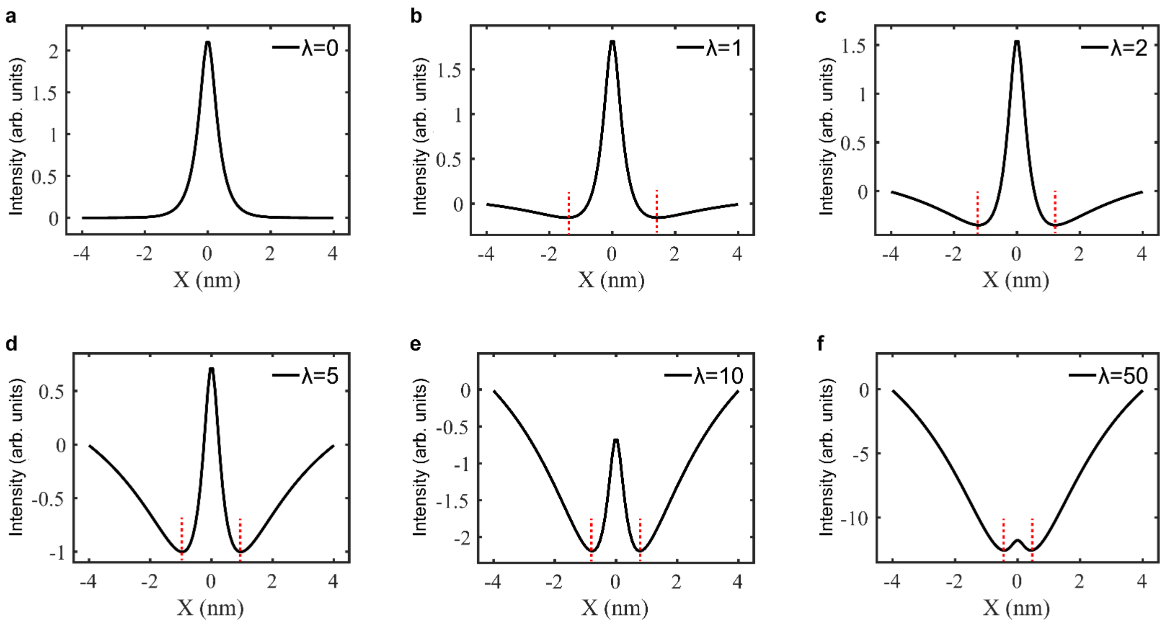

Supplementary Fig. 4: The spatial distribution of surface potential with increasing λ. (**a**) λ =0; (**b**) λ =1; (**c**) λ =2; (**d**) λ =5; (**e**) λ =10; (**f**) λ =50; The zero points of the horizontal and vertical axis correspond to the center of polaron and the potential surface away from the polaron. The red lines mark the position of shoulder peaks.

Supplementary Note. III *Ab initio* Simulation: Methodology and Results

To reveal the underlying physical mechanism and gain direct view of geometry and electronic structure of polaron in $CoCl_2$/HOPG, we perform *ab initio* calculations, and the simulation details are shown below.

(1) Structure relaxation methods

The polaron is studied by adding an excess electron on the CBM and relaxing the structure.

If we add one excess electron to optimized neutral $CoCl_2$ structure, we can directly obtain type-II polaron. However, for type-I polaron, we have generated initial distortion before structure optimization. First, we conjecture that the formation of polaron will elongate the bonds. Second, we set a parameter of polaron radius ($R_{polaron}$). Using one Cl or Co atom as the center, we draw a circle using $R_{polaron}$, elongate all the Co-Cl and Cl-Cl bonds in the circle by 10%, and use the distorted structure as the initial structure for polaron optimization. We have done the calculations using $R_{polaron}$ = 3, 4, 5, 6, 7 and 8 Å, as schematically shown in Supplementary Fig. 5. Using this approach, we successfully found type-I polaron when $R_{polaron}$ is 4 Å. Using this approach is easy to find the polarons with high symmetry.

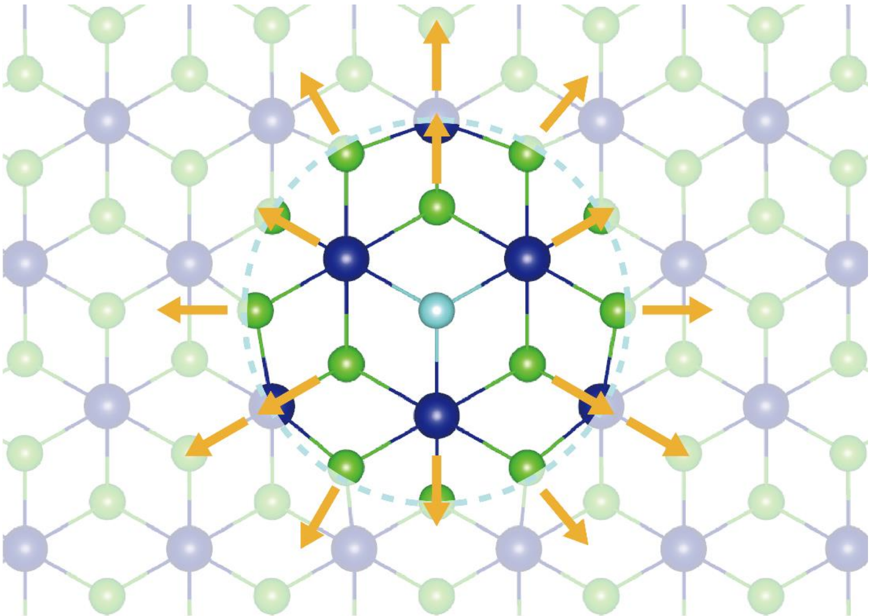

Supplementary Fig. 5: Initial distortion structure diagram. The dashed green circle represents the distortion range. The yellow arrows represent the directions of atom distortion. Purple and green balls represent cobalt and chlorine atoms, respectively. Sky blue ball represent distortion center chlorine atom.

(2) Polaron properties in monolayer $CoCl_2$

In order to have a comprehensive view of the structural distortion of polaron, we show the

main Co-Cl bond length changes of polaron I and II in Supplementary Table 1. For polaron I, the nine Co-Cl bonds in Supplementary Fig. 6 are all shorter. Because the d orbitals of the three Co atoms hybridize with each other, forming a bonding orbital, as shown in Fig. 4b, which shorten the distance of the three Co atoms, leading to the shortening of the Co-Cl bond. For polaron II, the six Co-Cl bonds around the central Co atoms are all elongate, which is consistent with the repelling surrounding anions properties of general electron polarons. In addition, the bond length changes for polaron I and polaron II are of the same magnitude. The large displacements in polaron I are mainly due to the bond angle changes.

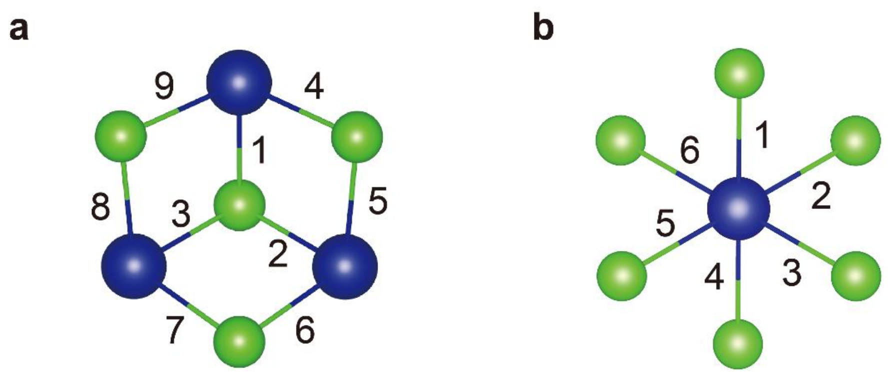

Supplementary Fig. 6: Structure distortion diagram of polaron I (a) and II (b). The numbers mark the main Co-Cl bonds in distortion.

Supplementary Table 1: The Co-Cl bond length changes during formation of polaron I and II. The bond labels are corresponded to those in Supplementary Fig. 6.

Co-Cl bond	1	2	3	4	5	6	7	8	9
Polaron I	-0.02	-0.03	-0.03	-0.03	-0.03	-0.03	-0.04	-0.04	-0.05
Polaron II	0.04	0.03	0.07	0.06	0.07	0.03			

In Supplementary Fig. 7a, we plot the spin-polarized projected density of states (PDOS) of

type II polaron. In addition to the polaron state that we labeled in Fig. 4c, there are several occupied states that are in the middle of the gap. They are separated from the valance band because of the lattice distortion. We plot the orbital distribution of the 5 mid-gap states shown in Supplementary Fig. 7 (b-f). They are mainly contributed by the 3*d* orbitals of Co atoms and localized within several unit cells.

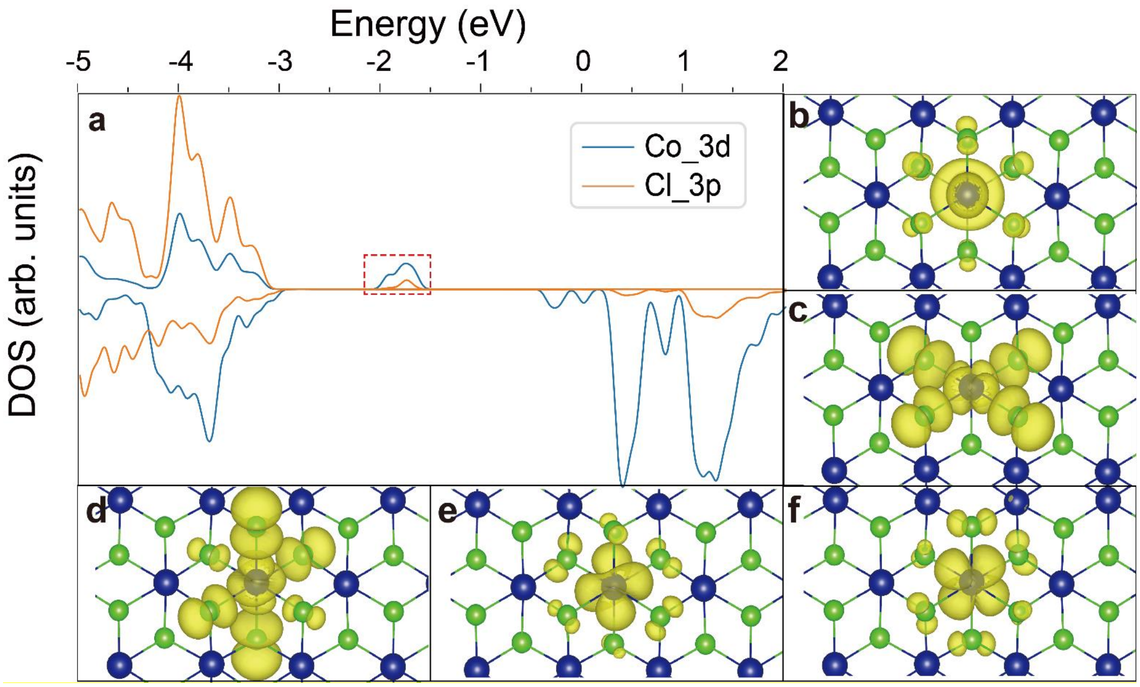

Supplementary Fig. 7: Density of states (DOS) of monolayer $CoCl_2$ with type II polaron (a) and the charge density distribution of the five mid-gap states (b-f). (a) The upper and lower parts represent spin up and down, respectively. The reference energy is the VBM energy. The red dotted rectangle marks the in-gap states.

To determine the radius of the polaron, we plot the displacement ($\Delta r_{\mathrm{i}} = \left|\overrightarrow{\mathbf{r}_{\mathrm{i}}} - \overrightarrow{\mathbf{r}_{\mathrm{i0}}}\right|$, $\overrightarrow{r_{i0}}$ and $\vec{r_i}$ are the coordinates of the i-th atom before and after the distortion) size of each atom, as shown in Supplementary Figure 8. We observe that the primary displacements are in close proximity, allowing us to encircle them with a circle. The circle's center is put on the center Cl atom and

Co atom for polaron I and II, respectively. We calculate the radii to be 5.4 Å and 3.5 Å for polaron I and II. The radius of polaron I is larger than polaron II, which is consistent with the previous experiments. In addition, we can analyze the radius of the polaron from the charge distribution. By summing up the proportion of charges in the sphere with different radii ($P = \iiint_{0\to R} |\psi|^2 r^2 \sin\theta dr d\theta d\varphi$, R is the radii, Ψ is the polaron state's wavefunction) and setting a threshold, such as 95%, we can obtain that the radius of the polaron charge distribution is 4.0 Å and 3.3 Å. This is similar to the radius of structural distortion.

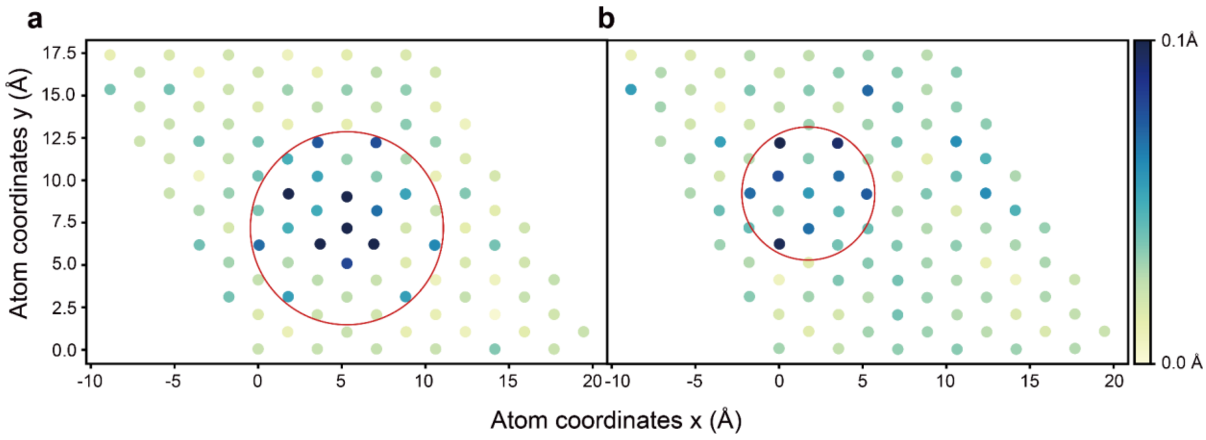

Supplementary Fig. 8: Schematic diagram of polaron I (a) and II (b) range. The small dots represent atoms. The colorbar shows the atomic displacement Δr_i during formation of polarons. The red circle corresponds to the polaron range.

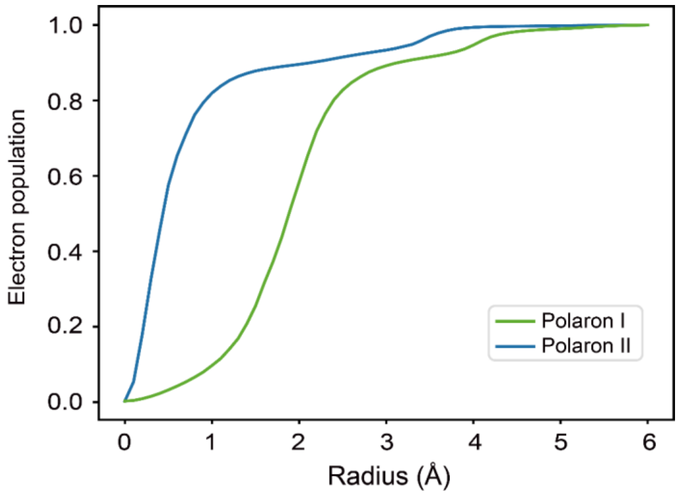

Supplementary Fig. 9: The summed Radial charge distribution (*P*) of polaron I and II.

(3) Polaron simulation in $CoCl_2$/graphite

In order to understand what does the graphite substrate affect the polaron nature of $CoCl_2$, we have added the graphite substrate and repeated the polaron calculation.

The $CoCl_2$/HOPG system was built by depositing a 4x4 supercell of monolayer $CoCl_2$ on a 6x6 supercell of 2 layers' graphite rather than 4-layers' graphite in order to reduce the computational cost. The results are shown in Supplementary Fig. 10. If one electron is added to the $CoCl_2$/graphite system, both type-I and type-II polarons can be maintained. In Supplementary Fig. 10(a, c), there is an in-gap state (labeled as Polaron I or II) for both two types of polarons. For polaron I, shown in Supplementary Fig. 10b, the charge distribution is distributed among three Co atoms around the central Cl atom, which is similar to the isolated $CoCl_2$. Same for Polaron II. According to the PDOS analysis, there is 85% and 70% electron density kept on $CoCl_2$, and 15% and 30% electron transfer to graphite for type-I and II polarons, respectively. Due to the small amount of charge transfer, the lattice distortion for both type-I and II polarons are smaller comparing with isolated $CoCl_2$, as shown in the left panels of Supplementary Fig. 10(b, d). But the orbital distribution characters are similar. The binding energies of type-I and II polarons are 287 and 307 meV, respectively. In summary, the graphite substrate does not change the major polaron properties, which shows the weak interaction between $CoCl_2$ layer and the graphite substrate.

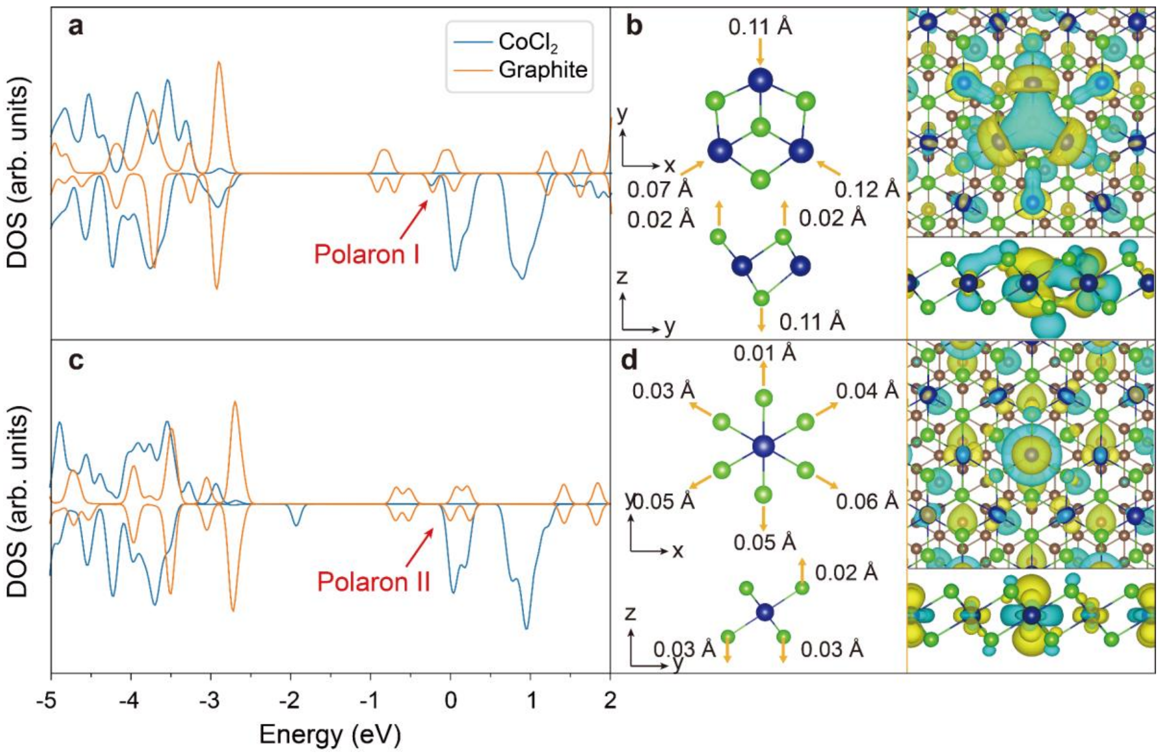

Supplementary Fig. 10: Polarons in $CoCl_2$/graphite. The up (**a**, **b**) and down panels (**c**, **d**) correspond to calculations of type-I and type-II polarons, respectively. (**a**, **c**) DOS of monolayer $CoCl_2$ with single polarons. The upper and lower parts represent spin up and down, respectively. The reference energy is CBM energy. The red arrow marks the polaron state. (**b**, **d**) Left panels: Structure distortion diagrams of $CoCl_2$/graphite induced by two types of polarons. The arrows represent the directions of atom distortion. Purple and green balls represent cobalt and chlorine atoms, respectively. Right panels: The charge density distribution (up panel: top view, down panel: side view) of two types of polarons.

In above $CoCl_2$/HOPG system, the lattice parameter of the $CoCl_2$/graphite heterostructure is optimized to be 3.69/2.46 Å. In this case, $CoCl_2$ has 4% tensile deformation. The 4% mismatch between $CoCl_2$ and graphite is large, but the interface strain is substantially relaxed by the weak interaction between $CoCl_2$ and graphite, as evidenced by the observation of various moiré patterns. In Supplementary Fig. 11, we have compared the band structure of free standing $CoCl_2$ with and without strain. One can see that their difference is negligible. Therefore, we propose that the graphite substrate does not change the major polaron conclusion.

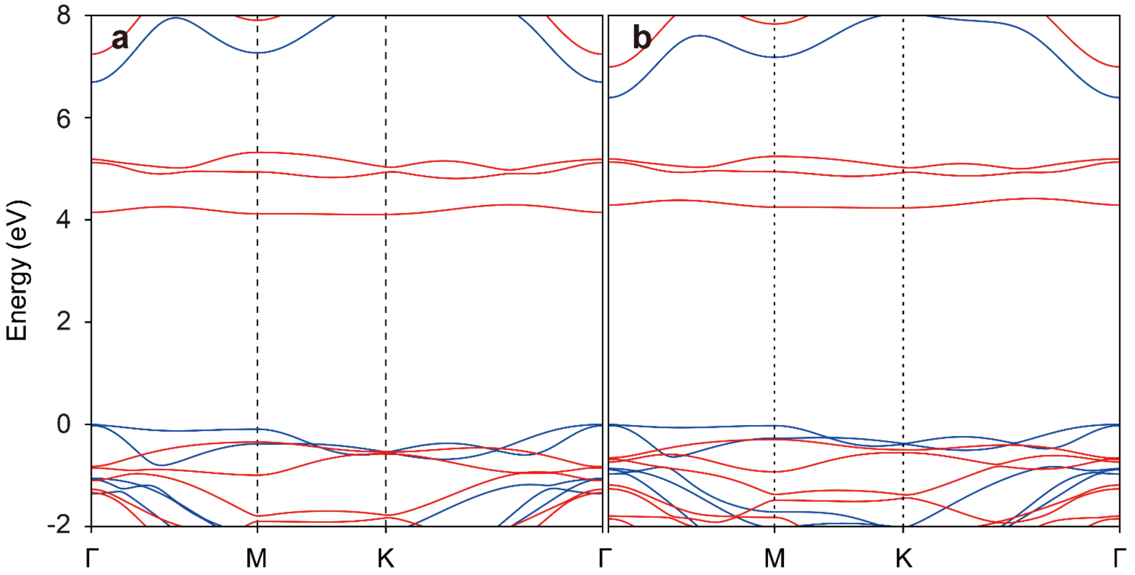

Supplementary Fig. 11: Band structure of free standing $CoCl_2$ without (a) and with (b) strain. The reference energy is the VBM energy. The blue and red bands correspond to spin up and down, respectively.

Supplementary Note. IV Hopping of polarons

Beside the manipulation processes like writing, erasure and transition applying on the polaron in $CoCl_2$ on HOPG, we also found the polaron can lateral hop to positions nearby by tip. Supplementary Figure 12 shows a series of STM images containing one polaron of $CoCl_2$ on HOPG with continuous scanning on same area. When the tip is close enough to the center of polaron, an instant hopping of polaron occurs on the surface. The step size of each movement should be an integer multiple of the atomic lattice period of $CoCl_2$.

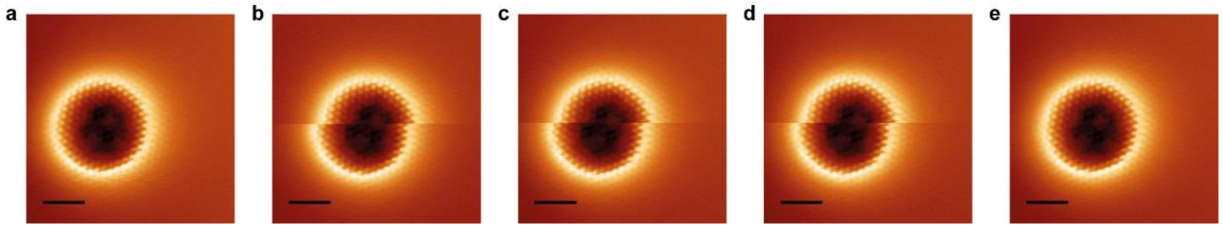

Supplementary Fig. 12: Lateral hopping of individual polaron. (a-e) A series of STM images of

monolayer $CoCl_2$ on HOPG with one polaron obtained by continuous scanning (V_s = 750 mV, I = 25 pA, V_{mod} = 20 mV). Scale bars: 2 nm. When the tip moves close to the polaron, it is possible induce the hopping of polaron.

In order to understand the hopping dynamics of polarons, we have performed first-principles simulations and use a linear interpolation method to calculate the hopping barriers between two type-I polarons and two type-II polarons. There are 20 interpolation points used between the initial and final structures. We also calculate the transition barrier between type-I and –II polarons, and the results are shown in Supplementary Fig. 13. The hopping barrier of type-I and -II polarons are 117 and 72 meV, respectively, and the transition barrier between type-I and type-II polarons is as small as 38 meV. Since linear interpolation method tends to overestimate the energy barriers, the real hopping and transition between these polarons are likely smaller, suggesting that the polaron hopping and transition events can easily occur at room temperature or under perturbations.

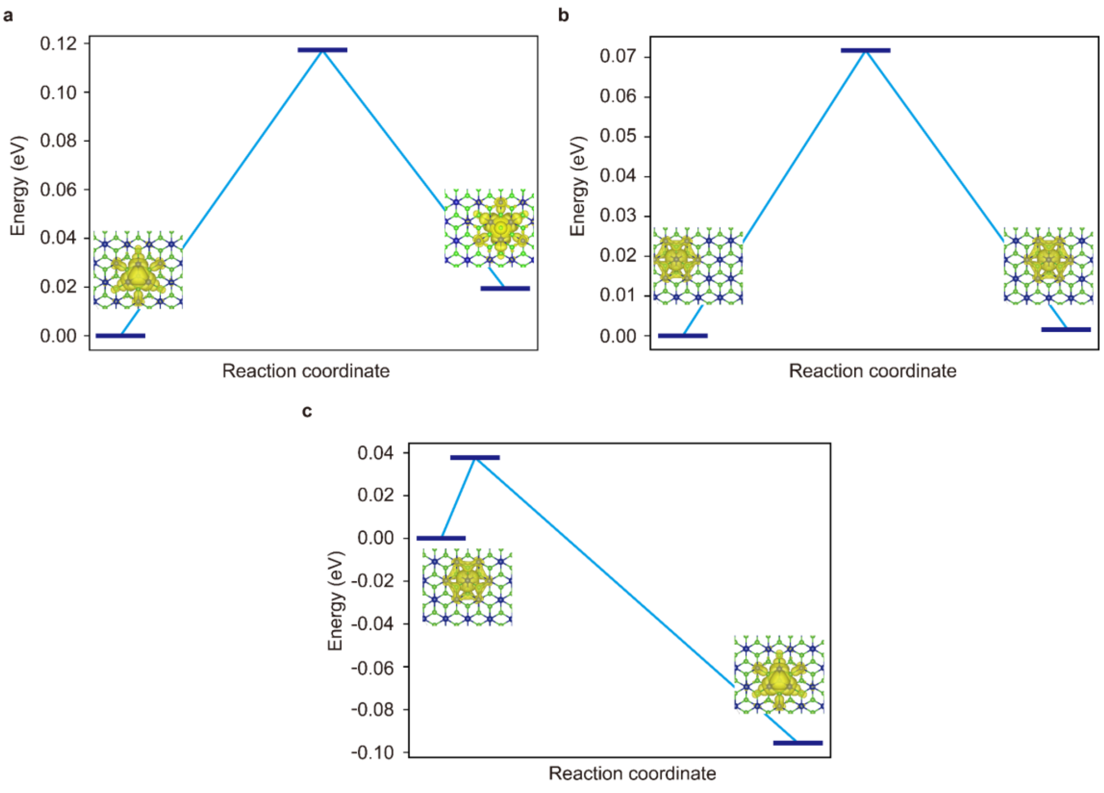

Supplementary Fig. 13: Hopping barriers between two type-I (a), type-II (b) polarons, and transition barrier between type-I and type-II polarons (c). The insets show the charge distribution of the initial and final polaron states. There are 20 interpolation points between the initial and final structures.

Supplementary Note. V Additional experiments and data

(1) The coexistence of different moiré patterns.

We have observed different moiré periodicities that are formed from the different orientation mismatch between the $CoCl_2$ layer and the HOPG substrate. For examples, Different $CoCl_2$ domains on the same upper layer of HOPG (Supplementary Fig. 14a) and one $CoCl_2$ layer spanning on adjacent domain with different orientations of HOPG (Supplementary Fig. 14b). The presence of different misorientations indicates the weak interaction between $CoCl_2$ layer and the HOPG substrate. It is worth highlighting that, we did not notice the influence of different moiré patterns on the property of polarons on the surface.

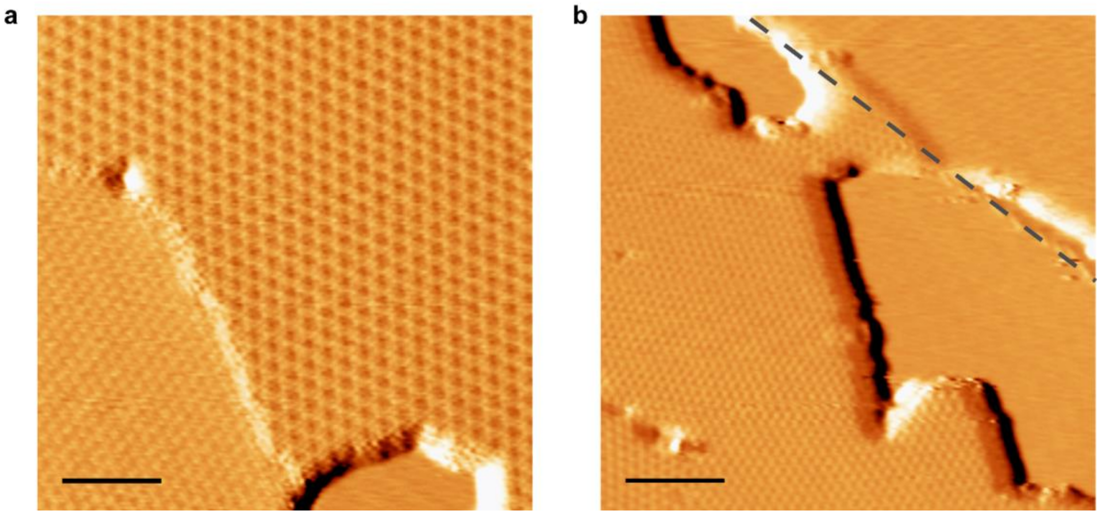

Supplementary Fig. 14: The coexistence of different moiré patterns of $CoCl_2$ monolayer on HOPG. (a) The derivative STM image of various moiré patterns formed by different $CoCl_2$ domains on the same layer of HOPG. (V_s = -0.5 V, I = 25 pA. Scale bar, 5 nm. **(b)** The derived STM image of various moiré patterns formed by $CoCl_2$ domains on adjacent area with different orientations of HOPG. The dotted line

marks the domain boundary of HOPG. (V_s = -1.0 V, I = 25 pA). Scale bar, 10 nm.

(2) The change of gap-size across different moiré patterns.

Supplementary Figure 15 shows the dI/dV curves taken on several $CoCl_2$ domains with different moiré periodicities. The gap-size is slightly different, which should come from the different coupling of $CoCl_2$ to the substrate. But as mentioned above, such difference may be not large enough to induce difference in polaron properties.

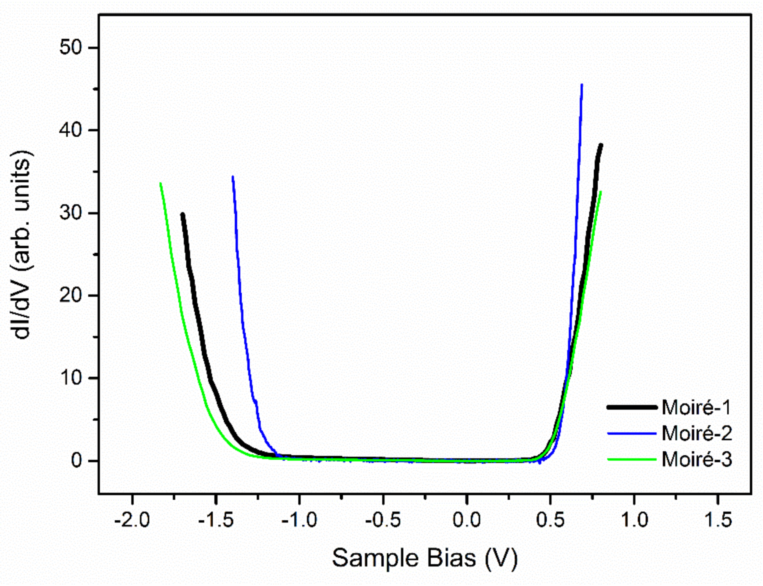

Supplementary Fig. 15: The d*I*/d*V* curves taken on several $CoCl_2$ domains with different moiré periodicities. The band gap is range of 1.6~1.8 eV under slightly different coupling to the substrate. The dI/dV spectrum highlighted by black bold is corresponding to Fig. 1e in the main text.

(3) **Batch writing and erasure of polarons**.

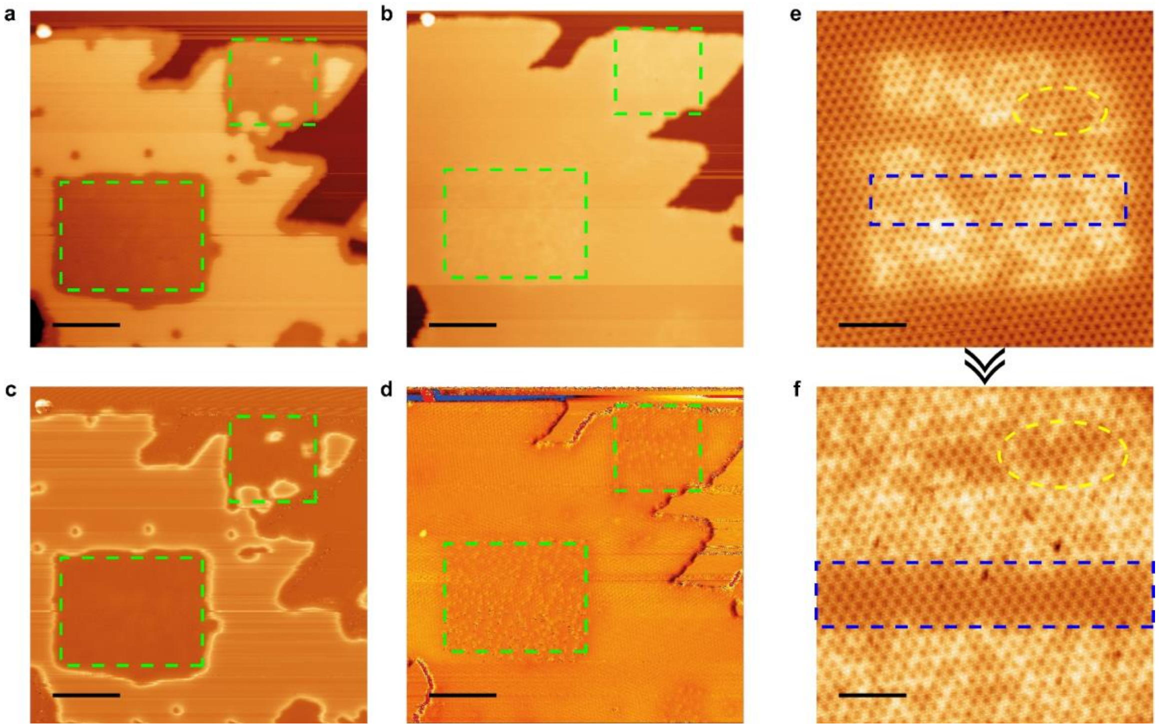

Supplementary Fig. 16: Batch writing and erasure of polarons. (a-d) The STM images and d*I*/d*V* maps taken on $CoCl_2$ monolayer on HOPG after continuous scanning with positive bias above 1 V. Green dotted rectangles mark the writing regions of 25 nm × 25 nm (upper right) and 45 nm × 40 nm (lower left). Scanning parameter: V_s = ±770 mV, I = 25 pA, V_{mod} = 20 mV with positive bias for **a, c** while negative bias for **b, d**. Scale bars, 20 nm. **(e-f)** STM images before and after collective erasion of polarons, respectively. The blue dotted rectangles represent the same area. And the yellow elliptical represents the collective nondirectional movements of positions during scanning with negative sample bias. Scanning parameter: V_s = 550 mV, I = 5 pA. Scale bars, 7 nm.

(4) In-gap states of polaron.

About in-gap states of polarons, no obviously polaron states were observed on STS measurement due to its extremely weak nature. As shown in Supplementary Fig. 17, with the decreasing of tip-sample spacing (tunneling current set from 5 pA to 25 pA), some weak in-gap states gradually appear in the background of the band bending. Supplementary Figure 17d

shows the contrast of the d*I*/d*V* spectrum obtained at the center of the polaron (black curve) and away from the polaron (red curve) when the tunneling current up to be 1 nA. This may be related to the intrinsic properties of polarons in $CoCl_2$/HOPG system.

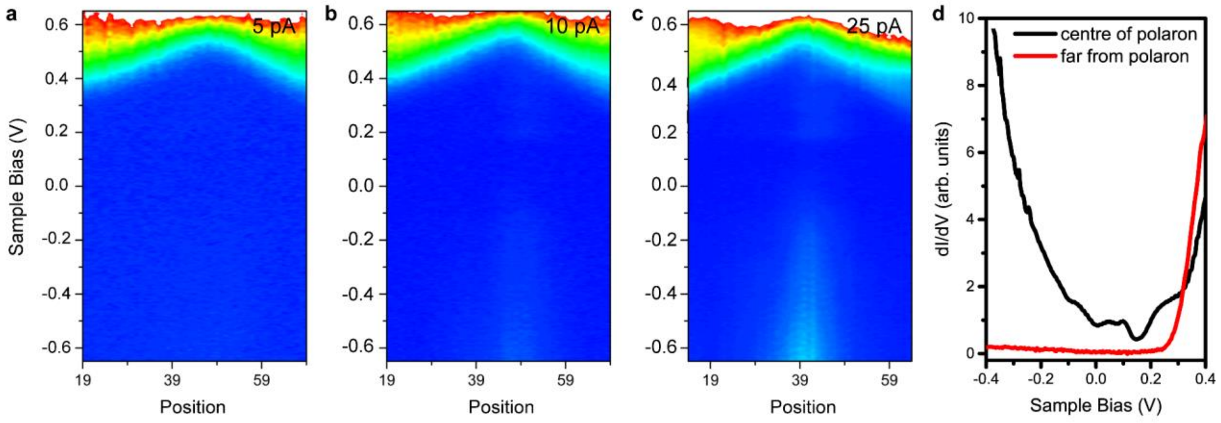

Supplementary Fig. 17: The in-gap features of the d*I*/d*V* spectra. (a-c) Line color maps across one polaron with the increased tunneling current, corresponding to 5 pA, 10 pA and 25 pA. (d) d*I*/d*V* curves with tunneling current of 1 nA. Black and red curves correspond to the spectrum at the center of the polaron and away from the polaron.

(5) Polarons on bilayer $CoCl_2$.

Increasing the coverage of $CoCl_2$ on HOPG, the second layer of $CoCl_2$ forms at the terrace of the monolayer island (Supplementary Fig. 18a), indicating the growth of $CoCl_2$ on HOPG is not the perfect layer-by-layer mode. Supplementary Figure 18b is the d*I*/d*V* curve showing the LDOS of $CoCl_2$ bilayer on HOPG. We can observe a larger band gap (~2.9 eV) than that of monolayer (~1.7 eV), which is closer to the bulk [11,12]. If we take the d*I*/d*V* measurements at bias voltage range within the bandgap, only flat and weak signal is observed, which is quite different from the V-shape feature observed on monolayer, indicating the tunneling electrons cannot directly penetrate the bilayer $CoCl_2$ from tip to HOPG or vice versa. Remarkably, the

polarons are also observed on bilayer $CoCl_2$. And different types appear as shown in Supplementary Fig. 18c. The polaron on bilayer $CoCl_2$ can also be manipulated by tunneling electrons, and one example of the polaron diffusion is shown in Supplementary Figs. 18d and 18e. The observation of polarons in monolayer and bilayer $CoCl_2$ implies the existence of stable polarons should be the intrinsic properties of $CoCl_2$ from 2D form to bulk.

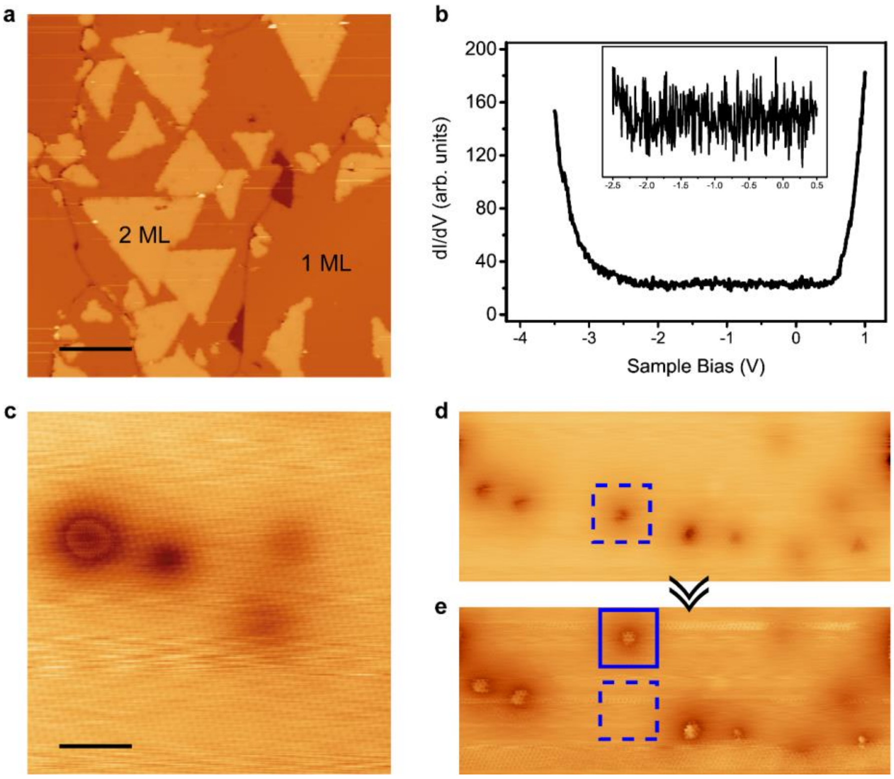

Supplementary Fig. 18: Polarons on bilayer $CoCl_2$. (a) STM image of large area with the coexistence of $CoCl_2$ bilayer and monolayer as labelled as “2 ML”, ”1 ML”. (250 nm × 250 nm, V_s = 1.2 V, I = 5 pA). Scale bar, 50 nm. **(b)** dI/dV curves taken on $CoCl_2$ bilayer with bias range from -3.5 V to +1.0V. A typical bandgap of about 2.9 eV is observed. The inset is the dI/dV curve with bias range from -2.5 V to +0.5 V. **(c)** STM image of $CoCl_2$ bilayer with polarons (V_s = 1.3 V, I = 5 pA). Scale bar, 3 nm. **(d-e)** STM images of same area of $CoCl_2$ bilayer before and after one polaron moves. Dotted and solid blue rectangles mark the change of the polaron position (32 nm × 15 nm, V_s = 1.9 V, I = 5 pA).

(6) **Monolayer $CoCl_2$ on Au(111) substrate.**

In general, HOPG has a weak coupling with 2D materials grown on it. This is found to be crucial to the existence of polaron in our system. For comparison, we grew $CoCl_2$ monolayer on Au(111) substrate (Supplementary Fig. 19a). The herringbone feature from Au(111) surface can be observed on monolayer $CoCl_2$ islands, indicating strong interactions between $CoCl_2$ monolayer and Au(111) substrate. The bias-depended STM image (Supplementary Figs. 19b and 19c) shows the similar unit cell of $CoCl_2$ as that on HOPG. However, we cannot observe any features related to the polarons. The absence of polarons may result from the strong interactions between $CoCl_2$ and Au(111), thus electrons can easily escaping to Au(111) substrate before being trapped by the monolayer $CoCl_2$.

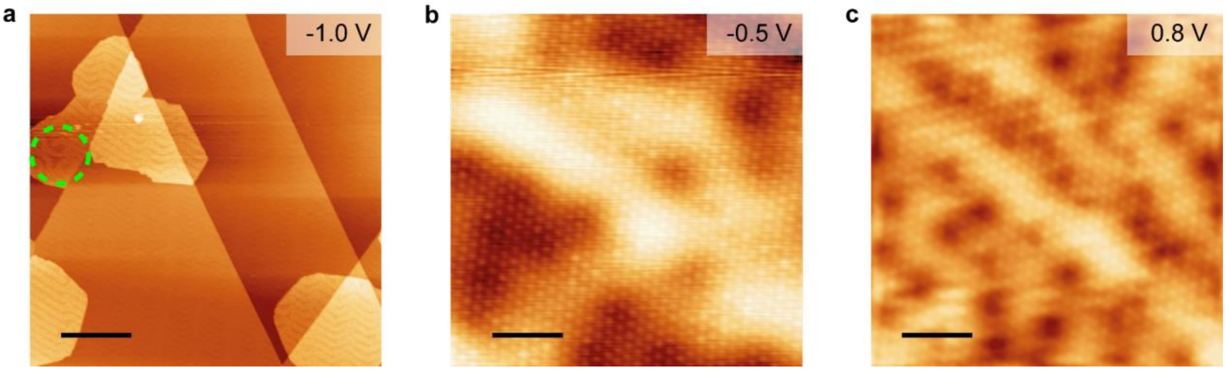

Supplementary Fig. 19: $CoCl_2$ monolayer on Au(111) substrate. (a) STM image of large area with $CoCl_2$ monolayer islands on Au(111) surface (V_s = -1 V, I = 10 pA). The herringbone-like feature with local slightly distortion (labelled by dotted circle) can be clearly observed on the surface of $CoCl_2$ monolayer, reflecting the strong interactions between Au and $CoCl_2$. Scale bar, 68 nm. **(b-c)** High-resolution STM images of same area on $CoCl_2$ monolayer on Au(111). Scanning parameters: V_s = -500 mV, I = 50 pA (**c**) and V_s = 800 mV, I = 5 pA (**d**). Scale bars, 2 nm.

Supplementary Note. VI Discussion of possible bi-polaron or polaron clustering

The interaction between polarons is a key factor in the operation and application of polarons, which involves two effects [13,14]: the direct Coulomb repulsion between like-charged

quasiparticles, and the interference of their atomic deformation patterns. If the interference energy gain is larger than the Coulomb repulsion, two polarons can be glued together forming a bi-polaron. If the relative strength between the interference energy gain and Coulomb repulsion is modest, there a room to allow two polarons to remain adjacent without gluing.

To gain a preliminary understanding of polaron interactions, especially during pattern construction or batching writing, we have focused on the distribution of the spacing between polarons (the distance between geometric centers). Statistical analysis shows that the closest distance between two polarons is roughly about 6 Å, as shown in Supplementary Fig. 20, which coincides with the typical sizes of polarons. Combined with the simulated results of stability of two adjacent polarons, we indicate that bi-polaron are absent in this specific system.

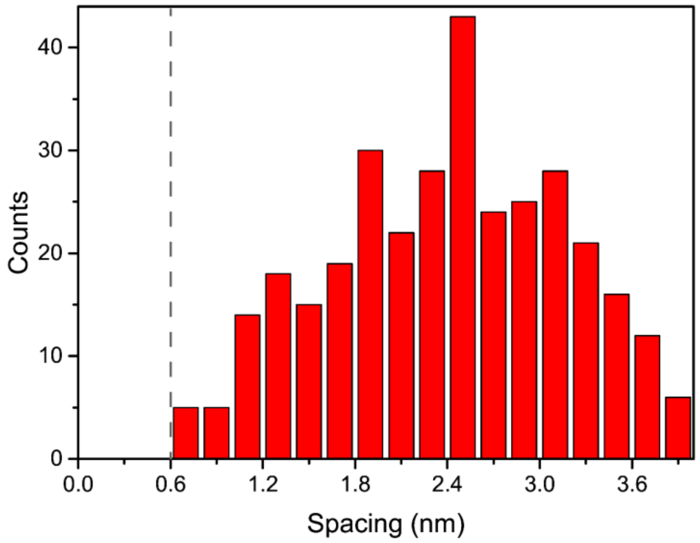

Supplementary Fig. 20: The statistical histogram of polaron spacing. Most polarons with spacing greater than 4.0 nm were not included considering the Coulomb potential range of about 3.0 nm. The total count and bin size is 331 points and 0.3 nm, respectively. The closest distance (dotted gray line) between polarons in all statistical data is found to be about 0.6 nm.

To get more insight of the physics, we simulate the stability of two polarons at different distances. For type-I polaron, we put two polaron’s distortions in the monolayer $CoCl_2$, with

the distances between the centers of the two distortions ($D_{polaron}$) being 12.0, 9.0, 6.0 and 3.5 Å, as shown in Supplementary Fig. 21. Due to the periodicity of the lattice, the two smallest $D_{polaron}$ are 3.5 and 6.0 Å. After conducting a structural relaxation with two excess electrons, we find the two type-I polarons can stabilize at $D_{polaron}$ =12.0, 9.0, 6.0 Å. When $D_{polaron}$ = 3.5 Å, the two electrons repel each other and one polaron move away from initial position. This suggests that the minimum distance between two polarons required for stable existence is 6.0 Å, which is consistent with the experiment result. The additional energy consumption to form two polarons is:

$$\Delta E = E_2 - (E_0 + 2(E_1 - E_0)) \tag{4}$$

Where E_2, E_1 and E_0 is the total energy of $CoCl_2$ system with two polarons, one polaron and no excess electron. The results are about 0.6 to 0.9 eV.

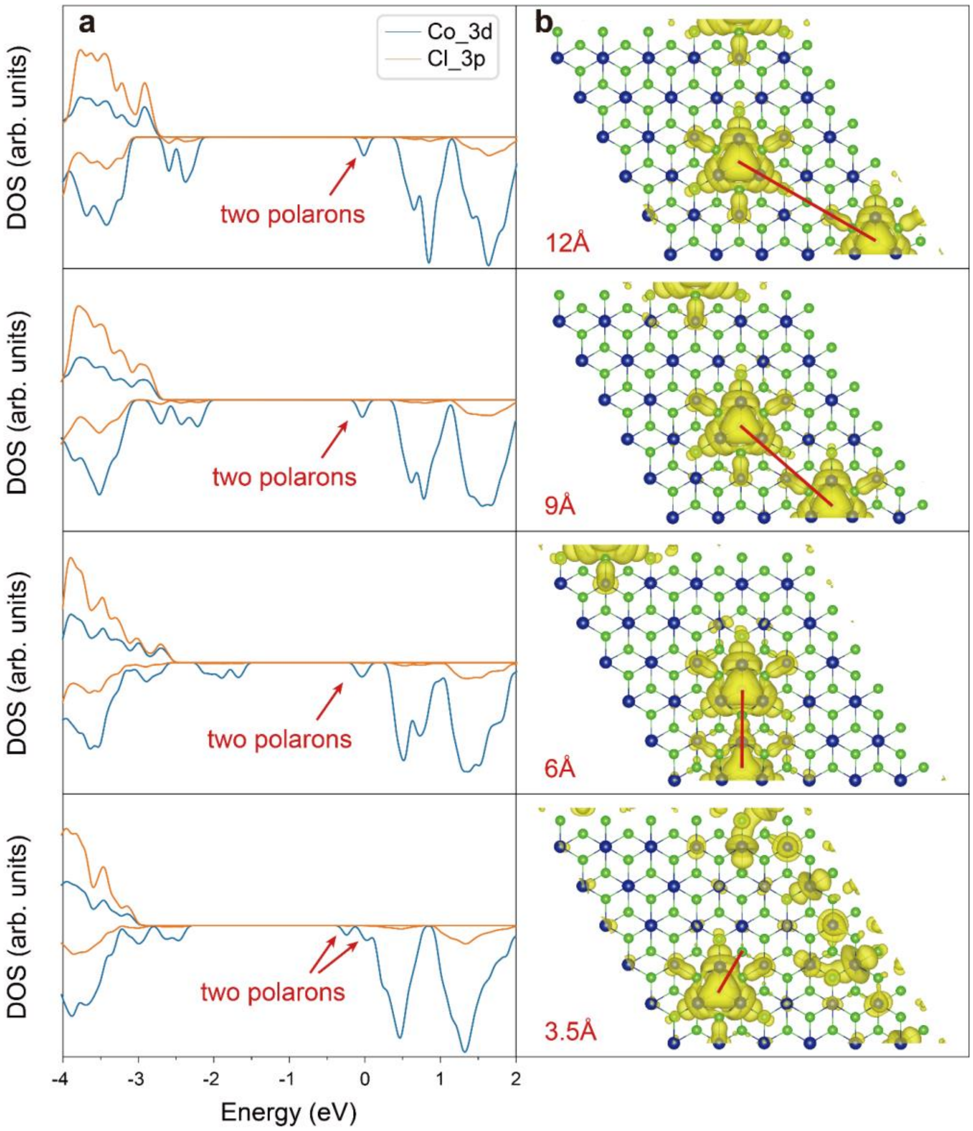

Supplementary Fig. 21: Modeling two polarons in monolayer $CoCl_2$. DOS (**a**) and charge distribution (**b**) of two polarons with different initial distortion distance 12.0, 9.0, 6.0, 3.5 Å. The reference energy is the VBM energy. The red short line marks the initial distortion center spacing. Purple and green balls represent cobalt and chlorine atoms, respectively.

Supplementary References:

1. Le Quang, T. et al. Band-bending induced by charged defects and edges of atomically thin transition metal dichalcogenide films. *2D Mater.* **5**, 035034 (2018).

2. Jena, D. & Konar, A. Enhancement of carrier mobility in semiconductor nanostructures by dielectric engineering. *Phys. Rev. Lett.* **98**, 136805 (2007).

3. Hanamura, E., Nagaosa, N., Kumagai, M. & Takagahara, T. Quantum wells with enhanced exciton effects and optical non-linearity. *Mate. Sci. Eng. B* **1**, 255-258 (1988).

4. Gan, Z., Xing, X. & Xu, Z. Effects of image charges, interfacial charge discreteness, and surface roughness on the zeta potential of spherical electric double layers. *J. Chem. Phys.* **137**, 034708 (2012).

5. Laubsch, A., Urban, K. & Ebert, P. Three- to two-dimensional transition in electrostatic screening of point charges at semiconductor surfaces studied by scanning tunneling microscopy. *Phys. Rev. B* **80**, 245314 (2009).

6. Teichmann, K. et al. Controlled charge switching on a single donor with a scanning tunneling microscope. *Phys. Rev. Lett.* **101**, 076103 (2008).

7. Wong, D. et al. Characterization and manipulation of individual defects in insulating hexagonal boron nitride using scanning tunnelling microscopy. *Nat. Nanotech.* **10**, 949-953 (2015).

8. Zheng, H., Kroger, J. & Berndt, R. Spectroscopy of single donors at ZnO(0001) surfaces. *Phys. Rev. Lett.* **108**, 076801 (2012).

9. Marczinowski, F., Wiebe, J., Meier, F., Hashimoto, K. & Wiesendanger, R. Effect of charge manipulation on scanning tunneling spectra of single Mn acceptors in InAs. *Phys. Rev. B* **77**, 115318 (2008).

10. Discovery of robust in-plane ferroelectricity in atomic-thick SnTe. *science* **353**, 274-278 (2016).

11. Fesefeldt, V. H. Weitere Absorptionsspektra chemisch einfacher Halogenidkristalle. *Z. Physik* **64**, 741-748 (1930).

12. Tubbs, M. R. The optical properties and chemical decomposition of halides with layer structures. *Phys. Stat. Sol.* **49**, 11-50 (1972).

13. Franchini, C., Reticcioli, M., Setvin, M. & Diebold, U. Polarons in materials. *Nat. Rev. Mater.* **6**, 560-586 (2021).

14. Emin, D. Small polarons. *Phys. Today* **35**, 34-40 (1982).
